# ISG15 regulates auto-inflammation by modulating NF-κB signaling pathway

**DOI:** 10.1016/j.gendis.2024.101462

**Published:** 2024-11-12

**Authors:** Meiqi Hou, Chao Yuan, Xiaopei Zhou, Zhenxing Liu, Nianyi Sun, Jinze Li, Xianqin Zhang

**Affiliations:** a Key Laboratory of Molecular Biophysics of the Ministry of Education, College of Life Science and Technology and Center for Human Genome Research, Huazhong University of Science and Technology, Wuhan, Hubei 430074, China; b Zhaoqing Medical College, Zhaoqing, Guangdong 526070, China; c Prenatal Diagnosis Center, Wuxi Maternity and Child Health Care Hospital, Wuxi, Jiangsu 214002, China

ISG15, the first identified ubiquitin-like protein stimulated by type I interferon, has multiple functions in the different vertebrate species and biological processes, such as anti-infection, autophagy, proliferation, cell death, and tumorigenesis.^1^ ISG15 has also been related to inflammation: human ISG15 deficiency results in necrotizing skin lesions through systemic type I IFN inflammation^2^; intracellular free ISG15 acts as a negative regulator of IFN-α/β-dependent autoinflammation by keeping USP18 stabilization.^3^ Despite these findings, the detailed molecular mechanisms how ISG15 regulates inflammation, especially neuroinflammation, remain largely elusive. This study used human microglia (HM cell line) and astrocytes (U87-MG cell line) to explore the molecular mechanisms underlying the association of neuroinflammation with ISG15 and find a negative regulatory mechanism of inflammation by ISG15. ISG15 post-translationally modified Ubc13, inhibiting the binding between Ubc13 and ubiquitin and preventing the K63 polyubiquitination of TRAF6, leading to the NF-κB signaling pathway inactive and then resulting in suppressed expression levels of pro-inflammatory cytokines and NLRP3. Furthermore, ISG15 positively regulated anti-inflammatory cytokines (IL-10, TGF-β, IL-35, IL-37, and IL-38) to prevent the expansion of the inflammatory response. Our finding suggests that ISG15 is a potential therapeutic target for inflammation.

To explore the role of ISG15 in inflammatory response, *ISG15* gene was silenced in the HM cells (Fig. 1A). When *ISG15* was knocked down, IL-1β and IL-6 rather than other inflammatory factors (TNF-α, IL-8, IL-18, and IL-1α) were significantly increased (Fig. 1A; Fig. S1A–C, 2A–D), suggesting ISG15 may negatively regulate the expression levels of IL-1β and IL-6. Using Western blot, the expression levels of intracellular IL-1β and IL-6 protein were also negatively regulated by ISG15 (Fig. S3). U87-MG cells are known for high background expression of IL-1β and low background expression of ISG15 (https://www.proteinatlas.org/), serving as a natural model to validate our finding. Similarly, overexpression of ISG15 in U87-MG cells significantly reduced the expression level of IL-1β and IL-6 (Fig. S1D–G, 2E–H). Meanwhile, using semi-quantitative reverse transcription-PCR analysis, the expression levels of anti-inflammation cytokines (IL-10, TGF-β, IL-35, IL-37, and IL-38) were significantly decreased following *ISG15* silencing in the HM cells (Fig. 1B; Fig. S1H–M). Correspondingly, in U87-MG cells, overexpression of ISG15 significantly increased the expression levels of anti-inflammation cytokines (Fig. S4). The secretion level of anti-inflammatory cytokines was positively regulated by ISG15 in both HM and U87 cells (Fig. S5). These data illustrate that ISG15 negatively regulates pro-inflammatory cytokines IL-1β and IL-6, while positively regulates anti-inflammatory cytokines (IL-10, TGF-β, IL-35, IL-37, and IL-38).

**Figure 1:**
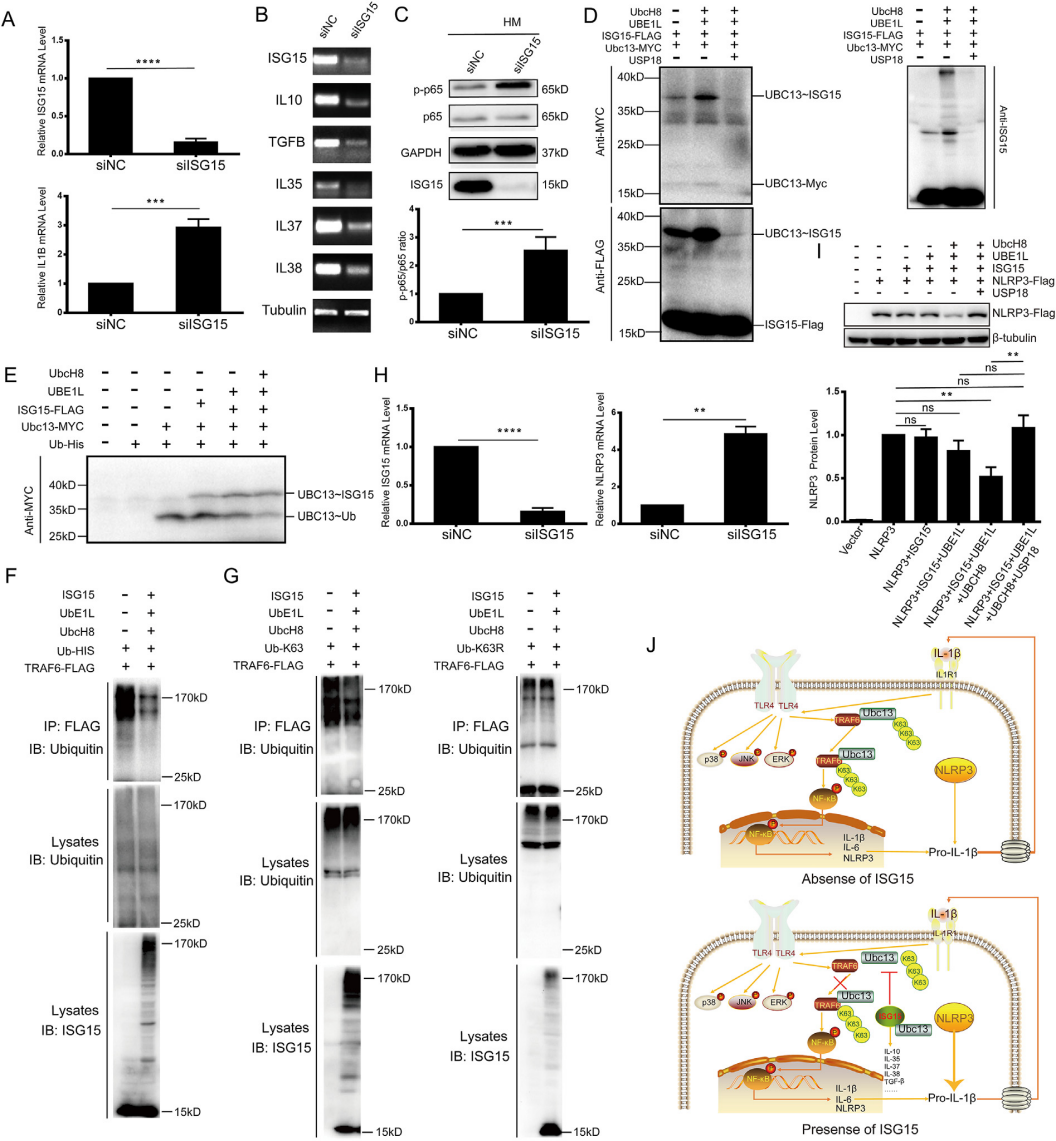
ISG15 negatively regulates inflammation through the NF-κB signaling pathway by ISGylating Ubc13. **(A)** Quantitative reverse transcription-PCR analysis of *ISG15* and *IL1B* in the HM cells. **(B)** Semi-quantitative RT-PCR analysis of *ISG15*, *IL10*, *TFGB*, *IL35*, *IL37*, and *IL38* in the HM cells with control siRNAs (siNC) and *ISG15* siRNA (siISG15). **(C)** Western blot analysis of p-p65, p65, and ISG15 between siNC and siISG15 in the HM cells. **(D)** Ubc13 was modified by ISGylation and USP18 protease could remove Ubc13 ISGylation. HEK293T cell extracts were immunoprecipitated with a FLAG antibody and analyzed by western blot using MYC, FLAG, and ISG15 antibodies. **(E)** Ubc13 ISGylation affected the thioester intermediate formation between Ubc13 and ubiquitin. **(F)** Western blot analysis of HEK293T cell lysates co-transfected with His-tagged ubiquitin, Flag-TRAF6, and the ISGylation system (UBE1L, UbCH8, and ISG15) after immunoprecipitation with anti-FLAG antibody. **(G)** Western blot analysis of HEK293T cell lysates when co-transfected with His-tagged K63 ubiquitin/K63R ubiquitin, Flag-TRAF6, and the ISGylation system (UBE1L, UbCH8, and ISG15) after immunoprecipitation with anti-FLAG antibody. **(H)** Quantitative reverse transcription-PCR analysis of *ISG15* and *NLRP3* in the HM cells transfected with negative control siRNA (siNC) and *ISG15* siRNA (siISG15). **(I)** ISGylation of Ubc13 decreased the expression level of NLRP3. Western blot analysis of HEK293T cell lysates when co-transfected with NLRP3-Flag and pcDNA3.1 (-), or NLRP3-Flag, and the ISGylation system with or without USP18. **(J)** The schematic diagram illustrating the negative regulatory mechanisms of ISG15 in inflammation. All quantitative reverse transcription-PCR experiments were repeated independently five times. All Western blot analysis was repeated independently three times. ∗∗∗∗*P* < 0.0001, ∗∗∗*P* < 0.001, and ∗∗*P* < 0.01; “ns” indicates no significant difference.

According to previous reports, the NF-κB signaling pathway plays a vital role in producing IL-1β.^4^ To explore whether ISG15 negatively regulates IL-1β via the NF-κB signaling pathway, immunoblotting and real-time quantitative PCR experiments were conducted to assess the expression level and activation of NF-κB. p-p65/p65 ratio was significantly increased after *ISG15* silencing in the HM cells, indicating enhanced activation of the NF-κB signaling pathway (Fig. 1C). Overexpression of ISG15 led to a decrease in the p-p65/p65 ratio in U87-MG and Hela cells (Fig. S6A–D). However, there was no significant difference in the mRNA level of NF-κB between siNC and siISG15 in the HM cells (Fig. S6F, G). Moreover, ISG15 did not affect the MAPKs (p-JNK, p-ERK1/2, and p-p38) signaling pathway in the HM cells (Fig. S6E). These data demonstrate that ISG15 specifically and negatively regulates IL-1β expression by modulating the activation of NF-κB.

Ubc13 has been reported to regulate the auto-ubiquitination of TRAF6 as a ubiquitin-conjugating E2 enzyme, subsequently leading to the activation of NF-κB.^5^ Co-immunoprecipitation experiments showed that Ubc13 interacted with ISG15 in the HEK293T cells, and detected two bands: the covalent band (∼36kD) and the free Ubc13 band (∼17kD) (Fig. S7A). The co-transfection of the ISGylation system (*ISG15*, *UBE1L*, and *UbcH8*) with Myc-Ubc13 indicated that Ubc13 could be modified by ISGylation (Fig. S7B). In addition, SDS-PAGE analysis revealed that Ubc13 bound to a single ISG15 molecule based on the size shift band (∼36kD). Similarly, we also verified the interaction between ISG15 and Ubc13 in the HM cells (Fig. S8). Moreover, USP18 (the specific deISGylation protease) could remove the ISGylation of Ubc13 (Fig. 1D). These results corroborate the modification of Ubc13 by ISGylation. Next, we investigated the functional effect of ISGylated Ubc13 and found that the formation of thioester intermediate between Ubc13 and ubiquitin was weakened when ISG15 and UBE1L were co-overexpressed, and even nearly disappeared when the ISGylation system (ISG15, UBE1L, and UbcH8) was overexpressed (Fig. 1E). All the above data demonstrate that Ubc13 is covalently modified by ISGylation, subsequently affecting the formation of thioester intermediate between Ubc13 and ubiquitin.

TRAF6 is known to generate K48- and K63-linked polyubiquitin chains, which may cause proteasomal degradation of target proteins or recruit target proteins for downstream signaling pathway transduction. Given Ubc13 ubiquitinated TRAF6 as a ubiquitin-conjugating E2 enzyme,^5^ we examined whether ISGylation affects the auto-ubiquitination of TRAF6. Our results indicated that the total auto-ubiquitination of TRAF6 was attenuated by ISGylation (Fig. 1F). Subsequently, we explored the specific type of ubiquitination of TRAF6 affected by Ubc13 ISGylation and detected that the level of K63-linked polyubiquitination of TRAF6 decreased when co-transfected with the ISGylation system (Fig. 1G). However, no change in the K48/K48R-linked polyubiquitination of TRAF6 was observed with or without the ISGylation system (Fig. S9). These results show that ISGylation of Ubc13 reduces the formation of thioester intermediate between Ubc13 and ubiquitin and affects the K63 polyubiquitination of TRAF6.

To further investigate the mechanistic connection between ISG15 and the negative regulation of IL-1β, the expression of inflammasome genes was examined using real-time quantitative PCR. The results showed that the mRNA expression level of *NLRP3* was increased after *ISG15* silencing in the HM cells (Fig. 1H) and decreased after Flag-ISG15 overexpression in U87-MG cells (Fig. S10A, B). Notably, the expression levels of other inflammasomes, including *NLRP1*, *NLRC4*, *AIM2*, and *IPAF*, remained unchanged between the siNC and siISG15 groups (Fig. S10C–F). These results suggest that ISG15 specifically regulates the expression of NLRP3 inflammasome. Subsequent examination of NLRP3 protein levels revealed a corresponding decrease upon the addition of the ISGylation system in the HEK293T cells (Fig. 1I). Moreover, the protein level of Flag-NLRP3 was decreased along with overexpressed ISG15 in a concentration-dependent manner (Fig. S10G). Remarkably, ISG15-specific protease USP18 could restore the reduced NLRP3 levels induced by Ubc13 ISGylation in a concentration-dependent manner (Fig. S10H). These results indicate that ISG15 negatively regulates NLRP3 expression at both the mRNA and protein levels.

Based on the data presented in this work, we propose a working model (Fig. 1J) to illustrate the process of ISG15-mediated inflammation regulation. In the absence of ISG15, Ubc13 serves as a ubiquitin-conjugating E2 enzyme and transfers K63-ubiquitin to TRAF6. Subsequently, phosphorylation modification of subunit p65 activates the transcription factor NF-κB, leading to significant increase of pro-inflammatory cytokines and NLRP3. While ISG15 is present, ISGylation post-translationally modifies Ubc13, inhibiting the binding between Ubc13 and ubiquitin and preventing K63 polyubiquitination of TRAF6. Consequently, the NF-κB signaling pathway remains inactive, resulting in suppressed expression levels of pro-inflammatory cytokines (such as IL-1β and IL-6) and NLRP3. Furthermore, ISG15 positively regulates anti-inflammatory cytokines (*e.g.*, IL-10, TGF-β, IL-35, IL-37, IL-38) to prevent the expansion of the inflammatory response. Thus, ISG15 may contribute to maintaining the M1/M2 balance of microglia by balancing proinflammatory and anti-inflammatory factors, thereby controlling excessive inflammation. In addition, the specific regulation of the NF-κB signaling pathway and NLRP3 inflammasome by ISGylation is more conducive to the precise development of new drugs for inflammation.

In conclusion, we found that ISG15 regulates auto-inflammation by negatively modulating the NF-κB signaling pathway through Ubc13 ISGylation modification. Therefore, ISG15 is a candidate therapeutic target for auto-inflammation in the brain.

## CRediT authorship contribution statement

**Meiqi Hou:** Conceptualization, Data curation, Formal analysis, Investigation, Methodology, Writing – original draft. **Chao Yuan:** Formal analysis, Investigation. **Xiaopei Zhou:** Formal analysis, Investigation. **Zhenxing Liu:** Formal analysis, Software. **Nianyi Sun:** Formal analysis, Investigation. **Jinze Li:** Formal analysis, Methodology. **Xianqin Zhang:** Conceptualization, Data curation, Funding acquisition, Investigation, Project administration, Resources, Supervision, Visualization, Writing – review & editing.

## Conflict of interests

The authors have no conflict of interests to report.
