# Supplementary material for: ISG15 regulates auto-inflammation by modulating NF-κB signaling pathway
Source: Genes Dis. 2024 Nov 12;12(4):101462. doi: 10.1016/j.gendis.2024.101462 (PMC11985037; doi:10.1016/j.gendis.2024.101462)
Supplement: Multimedia component 1 [file mmc1.docx]

**Materials and Methods**

**Construction of expression vector**

cDNA of ISG15 was constructed with 3×Flag-CMV10, and pcDNA3.1 (-) empty vectors, cDNA of UBE1L, UbcH8, USP18 were constructed with pcDNA3.1 (-), cDNA of TRAF6 was constructed with 3×Flag-CMV10, cDNA of Ubc13 was constructed with pCMV-MYC, cDNA of NLRP3 was constructed with 3×Flag-CMV10, using a Hieff Clone® Plus One Step Cloning Kit (Yeasen, Shanghai, China) according to the manufacturer’s instructions. Vectors of Ub, Ub-K63, Ub-K63R, Ub-K48, and Ub-K48R were given as gifts by professor Wuhan Xiao of Institute of Hydrobiology of Chinese Academy of Sciences. DNA sequence of vectors was confirmed by Sanger DNA sequencing.

**Cell culture and transfection**

HEK293T, Hela, human microglia (HM, ATCC, USA) and U87-MG (ATCC, USA) cells were cultured (37℃, 5% CO2) in Dulbecco's modified eagle medium (DMEM, Hyclone, USA) complemented with 10% fetal bovine serum (FBS, Invigentech, USA). Constructs and siRNA were transiently transfected into cultured cells with LipoFectMax™ 3000 Transfection Reagent (ABP Biosciences, the USA) according to the manufacturer’s instructions.

**RNA extraction and reverse transcription**

Total RNA was extracted from HM, U87-MG cells using TRIzol reagent (Takara, Japan) according to the manufacturer’s instructions and stored at -80°C. With total RNA as templet, cDNAs were obtained using GoldenstarTM RT6 cDNA Synthesis Mix (gDNA remover and Rnasin selected) (Tsingke, China) according to the manufacturer’s instructions and stored at -80°C.

**Semi-Quantitative real time-PCR (RT-PCR) and Quantitative real time-PCR (qRT-PCR)**

Product of reverse transcript cDNAs was performed qRT-PCR using AceQ qPCR SYBR Green Master Mix (Vazyme, China) according to the manufacturer’s instructions. β-actin was used as an internal loading control, and ΔΔCT variations were calculated. For semi-quantitative RT-PCR, cDNA was conducted PCR with specific gene primers (Table S1) and electrophoresis with 2% agarose gel (Tsingke, China). Following, images were showed in ChemiDoc XRS+ (BioRad, the USA) and gray value was scanned for further data analysis.

**Immunoprecipitation**

Constructs were co-transfected into cultured HEK293T cells with the LipoFectMax™ 3000 Transfection Reagent (ABP Biosciences, China). Protein was extracted with Western and IP lysis buffer (Beyotime, China) from harvested cells 36 hours after transfection. The primary antibody (Proteintech, China) was incubated with the cell lysis for immunoprecipitation, the control IgG (ABclonal, China) was incubated with a part of the cell lysis for as negative control overnight at 4°C. 100 μL cell lysis was input as positive control. Then, the protein A/G magnetic bead (MedChemExpress, USA) was incubated with the immunoprecipitated proteins for 4 hours at 4°C. The magnetic beads which was separated with magnetic grate was washed four times with PBS (Phosphate Buffered Saline, pH7.4). Then the samples were eluted from magnetic beads with 2×SDS-PAGE loading buffer (Boster, the USA) for further analysis.

**Western blot**

Cells were collected and lysed using cell lysis buffer (Beyotime, China) for 30 minutes at 4℃ and then centrifuged at 12000 g at 4℃. The supernatant was collected after 30 minutes. After combined with SDS-PAGE loading buffer (Reducing, 5×) (Cwbio, China) then proteins were heated at 100℃ for 10 minutes, and then 20 μg of protein was loaded in each well and were separated by electrophoresis on 12% SDS polyacrylamide gel followed by electroblotted on PVDF membranes (Millipore, USA) at 200 mA in the ice-water mixture. After transfer (30 minutes-2 hours), with 5% non-fat-dried milk (Becton, Dickinson and Company, USA), the background protein was obstructed. With anti-p-p65, p65, p-JNK, p-ERK1/2, p-p38 (Wanleibio, China), anti-ISG15, GAPDH, β-actin, MYC, FLAG (ABclonal, USA), anti-ubiquitin (Proteintech, China), the PVDF membranes was incubated for 12 hours at 4°C. After washed with TBST (Tris Buffered Saline with Tween 20), corresponding secondary antibody (Cwbio, China) was incubated for 2 hours at room temperature. Then outcomes were obtatined using West Pico PLUS Chemiluminescent Subs trat (Biology, China) with ChemiDoc XRS^+^ The membrane after repeated washed with TBST.

**ELISA**

The supernatant of HM (human microglia) cells was collected after 48 hours transfected and LPS treated (positive control, 1 μg/mL) and were determined using human IL-1β ELISA kit (Mlbio, China) according to the manufacturer's instructions.

**Statistical Analysis**

Statistical data are presented as mean ± SD for three or five independent experiments. Significant differences of mean values were performed by Student’s t test. All analysis was performed with GraphPad Prism (Version 6.01).


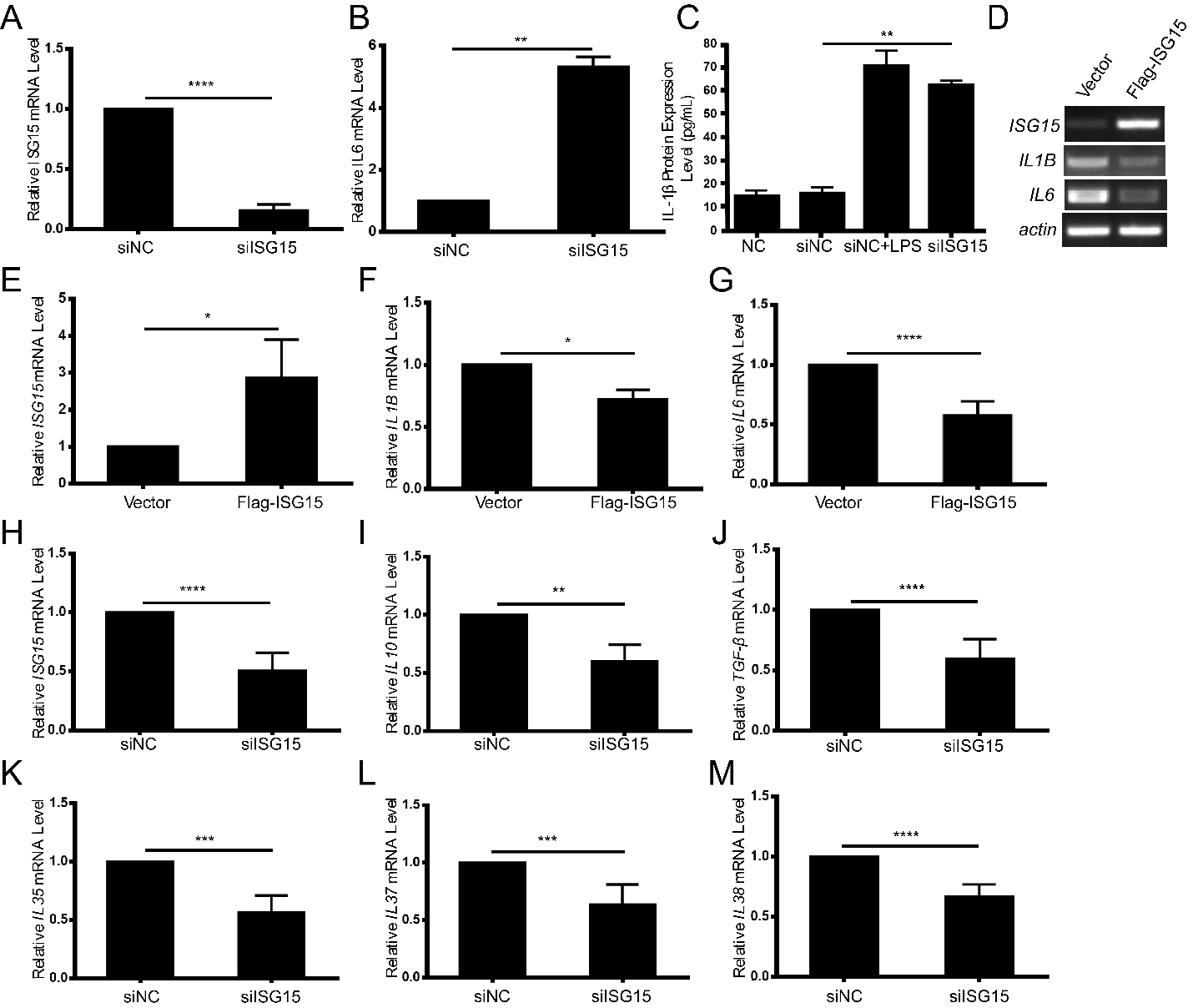


Fig. S1. ISG15 negatively regulates the expression levels of inflammatory factors in HM and U87 cells. (A-B) Quantitative RT-PCR analysis of *ISG15*, *IL6* gene in HM cells. (C) The statistical analysis of ELISA results of IL-1β after 48 h treatment with control siRNA (siNC) and *ISG15* gene siRNA (siISG15) in HM cells. The positive control (siNC+LPS) is treated with LPS (1μg/mL) for 12 h. (D) Semi-quantitative RT-PCR analysis of *ISG15, IL1B, IL6* gene in U87-MG cells with or without Flag-ISG15. (E-G) Statistical data analysis of *ISG15* (E), *IL1B* (F), *IL6* (G) expressions in U87-MG cells. (H-M) Statistical data analysis of semi-quantitative RT-PCR results of *ISG15* (H), *IL10* (I), *TFGB* (J), *IL35* (K), *IL37* (L) and *IL38* (M) in HM cells with control siRNAs (siNC) and *ISG15* gene silencing (siISG15). All semi-quantitative and quantitative RT-PCR experiments were repeated independently five times. Tubulin: internal loading control. Levels of significance: ****P < 0.0001, ***P < 0.001, **P < 0.01, *P < 0.05.


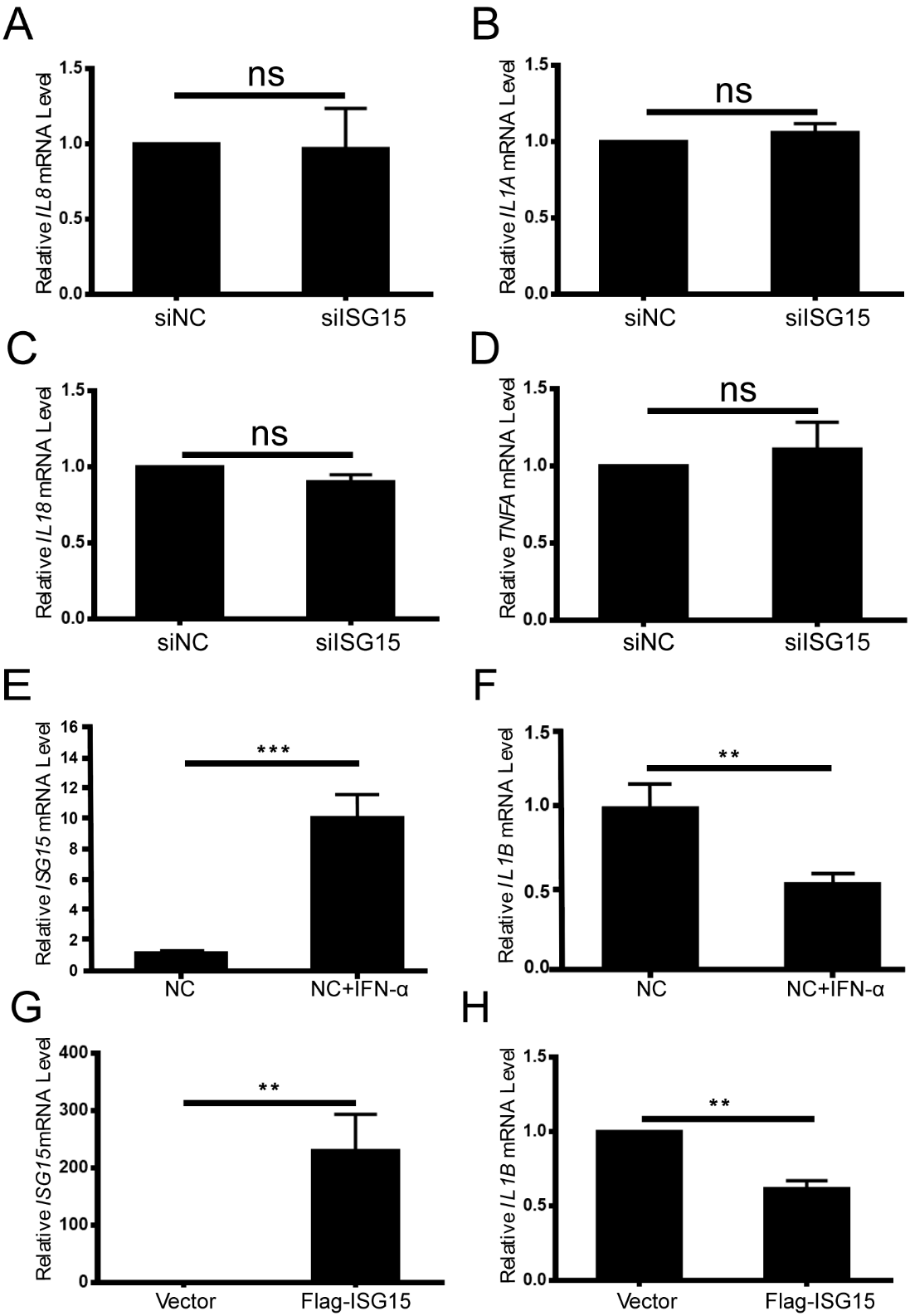


Fig. S2. The expression levels of pro-inflammatory factors were negatively regulated by ISG15. (A-D) Quantitative RT-PCR analysis of *IL8* (A), *IL1A* (B), *IL18* (C) and *TNFA* (D) gene. (E-F) Quantitative RT-PCR analysis of *ISG15* and *IL1B* gene between negative control and IFNα2b (10 ng/mL, SIGMA, Germany) for 6 h. (G-H) Quantitative RT-PCR analysis of *ISG15* and *IL1B* gene between negative control (Vector) and ectopic expression of *ISG15* (Flag-ISG15). All quantitative RT-PCR experiments are repeated independently five times. Levels of significance: “ns” stands for no significant difference， ***P < 0.001, **P < 0.01.


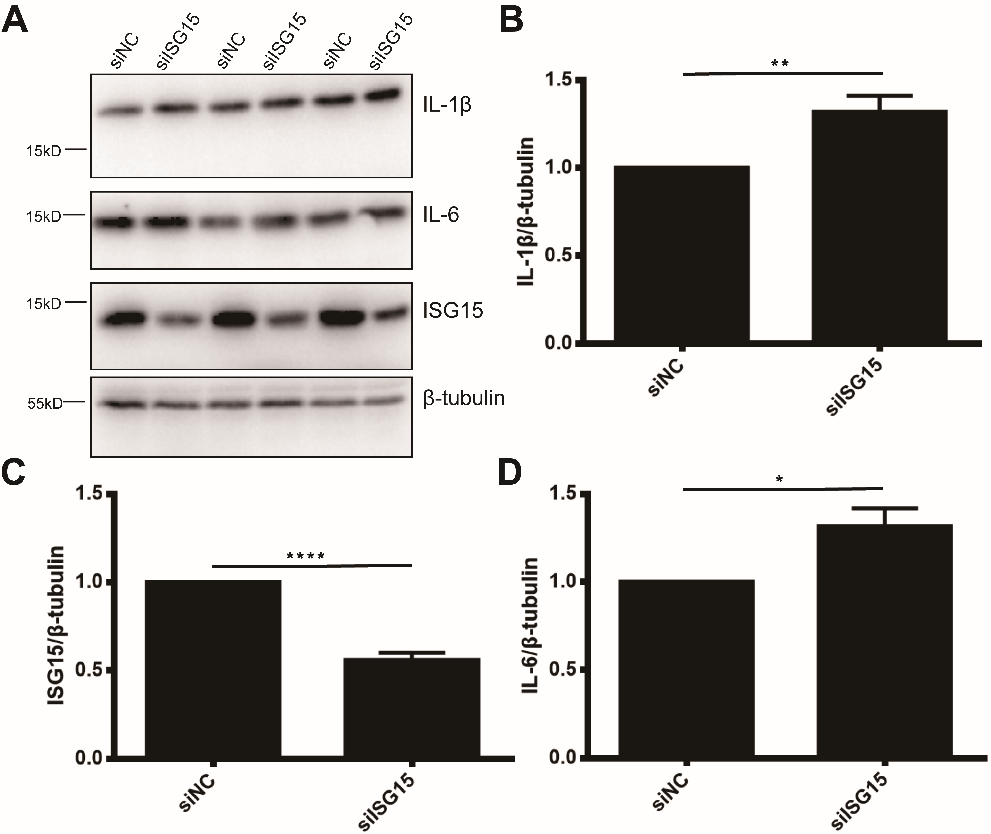


**Figure S3. The expression level of IL-1β and IL-6 protein was increased when ISG15 silencing in the HM cells. (A)** Western blot analysis of and IL-1β, IL-6, ISG15 in HM cells. **(B-D)** The statistical result of (A). β-tubulin was used the loading control. All western blot analysis was repeated independently three times. Levels of significance: *P < 0.05, **P < 0.01, ****P < 0.0001.


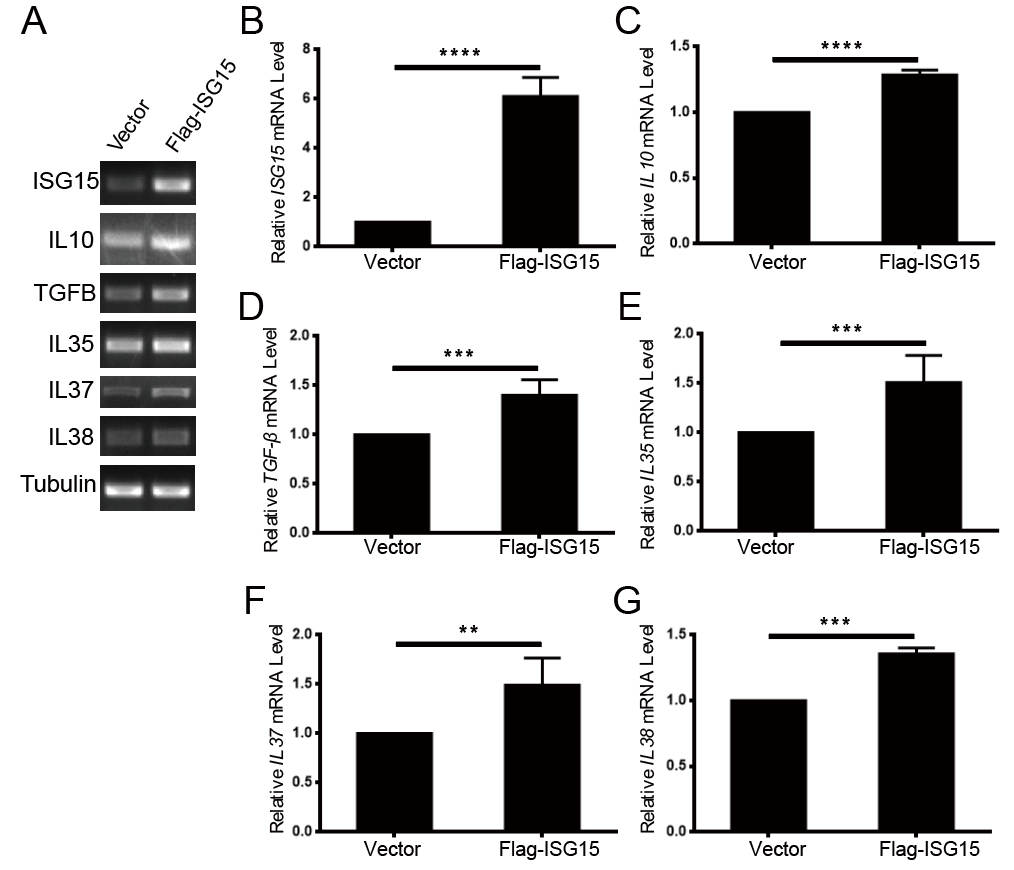


Figure S4. The expression levels of anti-inflammatory factors are increased by overexpressed ISG15 in U87 cells. (A) Semi-quantitative RT-PCR analysis of *ISG15*, *IL10*, *TGFB*, *IL35*, *IL37* and *IL38* gene between vector and Flag-ISG15. (B-G) The statistical data analysis of *ISG15* (B), *IL10* (C), *TGF-β* (D), *IL35* (E), *IL37* (F) and *IL38* (G). All Semi-quantitative RT-PCR experiments are repeated independently three times. Levels of significance: ****P < 0.0001, ***P < 0.001, **P < 0.01.


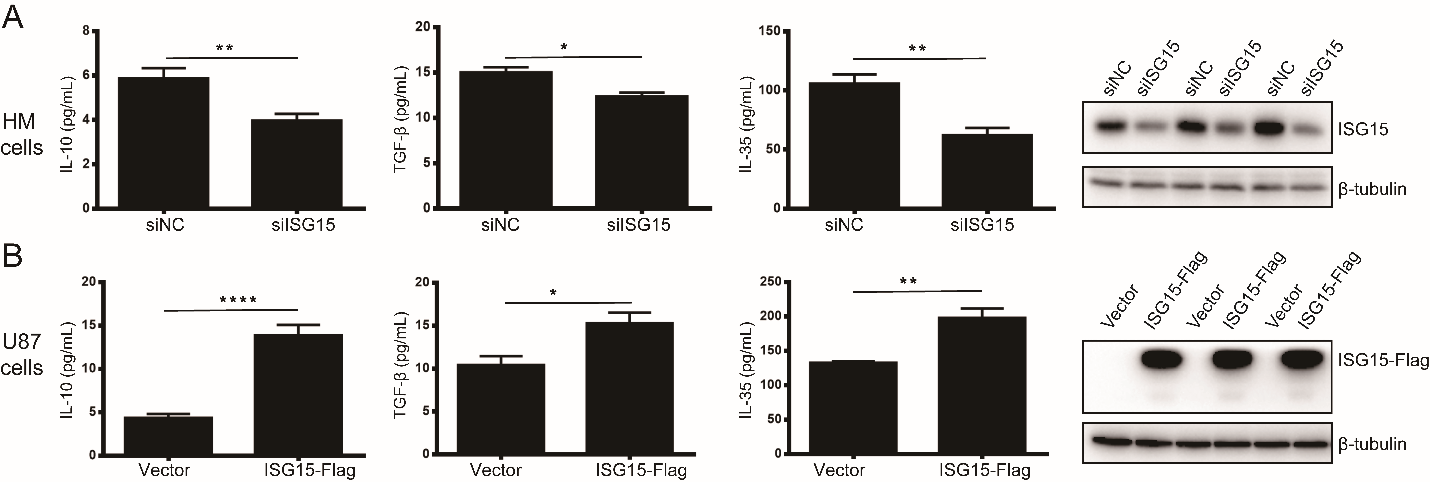


Figure S5. ISG15 positively the secretion level of anti-inflammatory cytokines both in HM and U87 cells. (A) ELISA analysis of IL-10, TGF-β, IL-35 in HM cells. (B) ELISA analysis of IL-10, TGF-β, IL-35 in U87 cells. All ELISA experiments were repeated independently three times and all western blot analysis are repeated independently three times. Levels of significance: *P < 0.05, **P < 0.01, ****P < 0.0001.


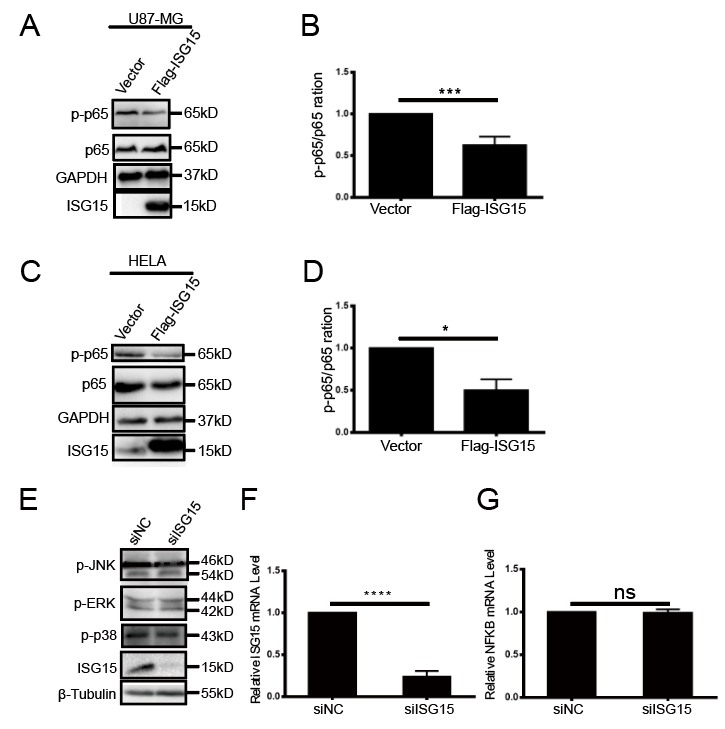


Figure S6. ISG15 negatively regulates IL-1β expression through the NF-κB signaling pathway. (A, B) Western blot analysis of p-p65, p65 and ISG15 in U87-MG cells. (C, D) Western blot analysis of p-p65, p65 and ISG15 in HELA cells. (E) Western blot analysis of p-JNK, p-ERK, p-p38 and ISG15 in HM cells. (F, G) Quantitative RT-PCR analysis of *ISG15* (F) and *NFKB* (G) gene. GAPDH and β-tubulin was used the loading control. All quantitative RT-PCR experiments were repeated independently five times and all western blot analysis are repeated independently three times. Levels of significance: *P < 0.05, ***P < 0.001, ****P < 0.0001, “ns” stands for no significant difference.


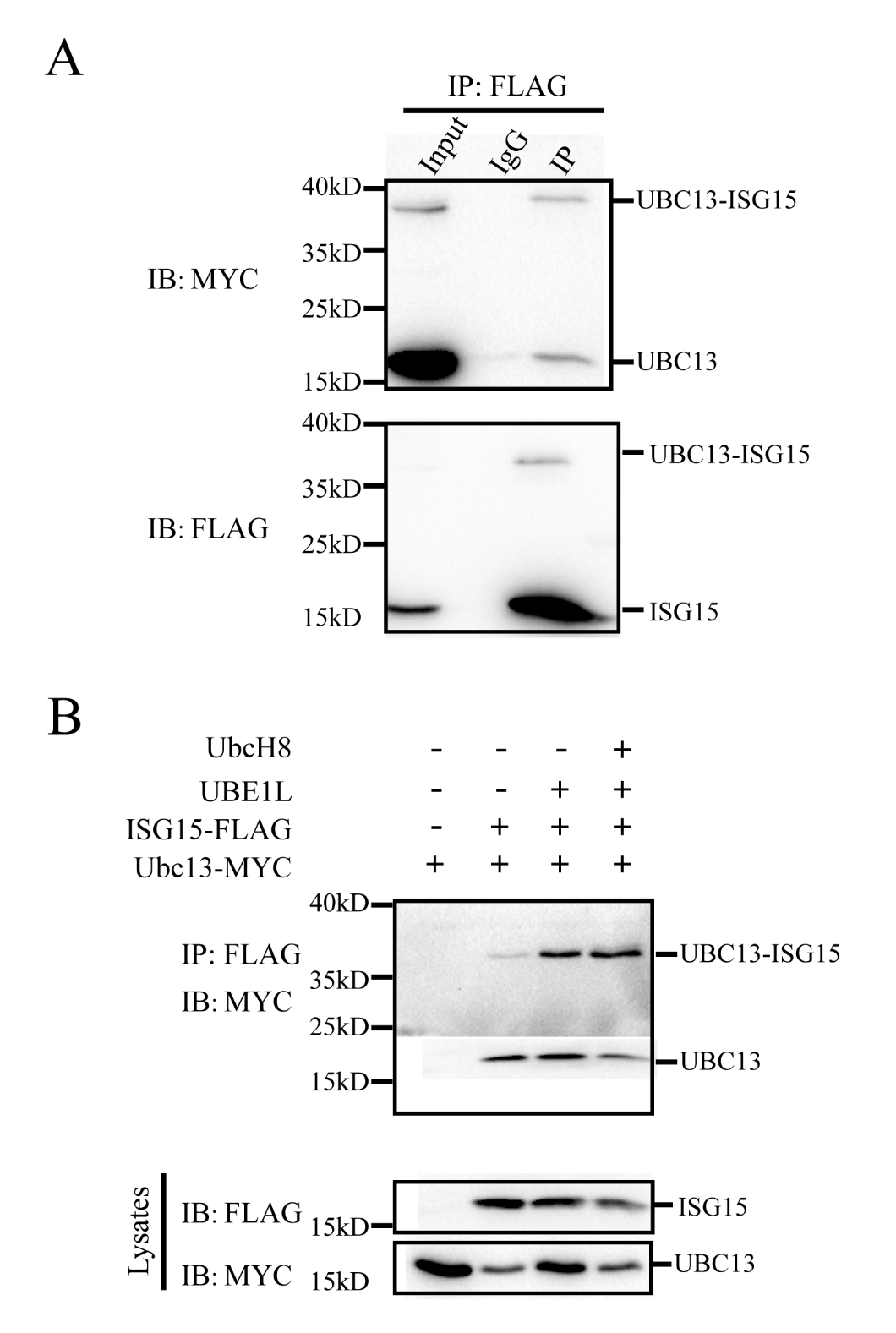


Figure S7. ISG15 negatively regulates NF-κB signaling pathway by ISGylating Ubc13. (A) Co-immunoprecipitation of Flag-ISG15 and Myc-Ubc13 analyzed by western blot. Input: cell lysate; IgG: negative control; IP-FLAG: immunoprecipitation using FLAG antibodies; IB-MYC, Myc-Ubc13; IB-FLAG, Flag-ISG15. (B) Ubc13 is modified by ISGylation. HEK293T cell extracts are immunoprecipitated with a Flag antibody and analyzed by Western blot using MYC and FLAG antibodies.


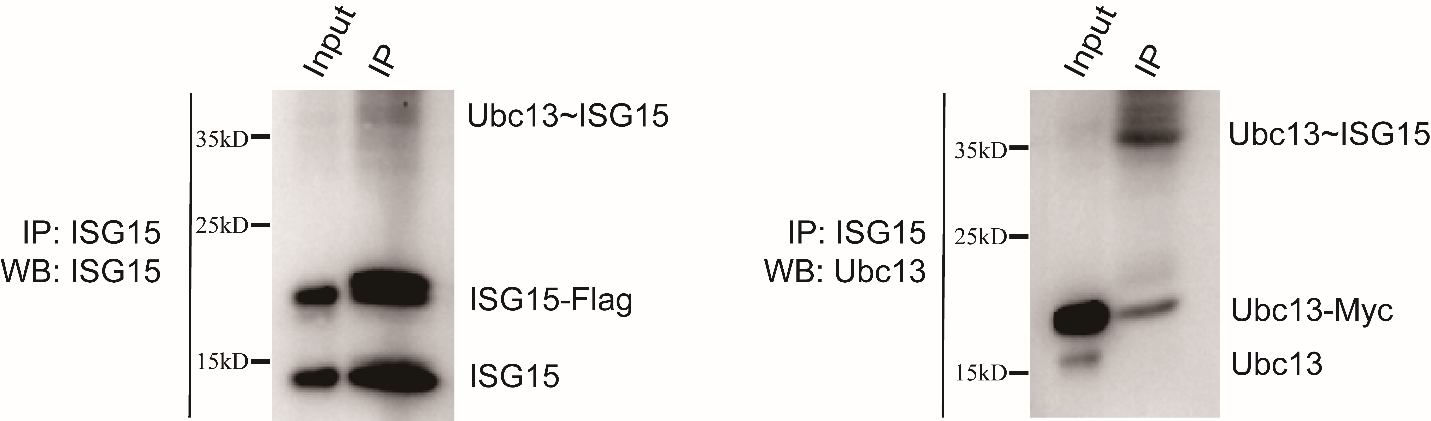


Figure S8. The interaction between ISG15 and Ubc13 in HM cells. Co-immunoprecipitation of Flag-ISG15 and Myc-Ubc13 analyzed by western blot. Input, cell lysate; IgG, negative control; IP: ISG15, immunoprecipitation using ISG15 antibodies; IB: ISG15, western blot using ISG15 antibodies; IB: Ubc13, western blot using Ubc13 antibodies.


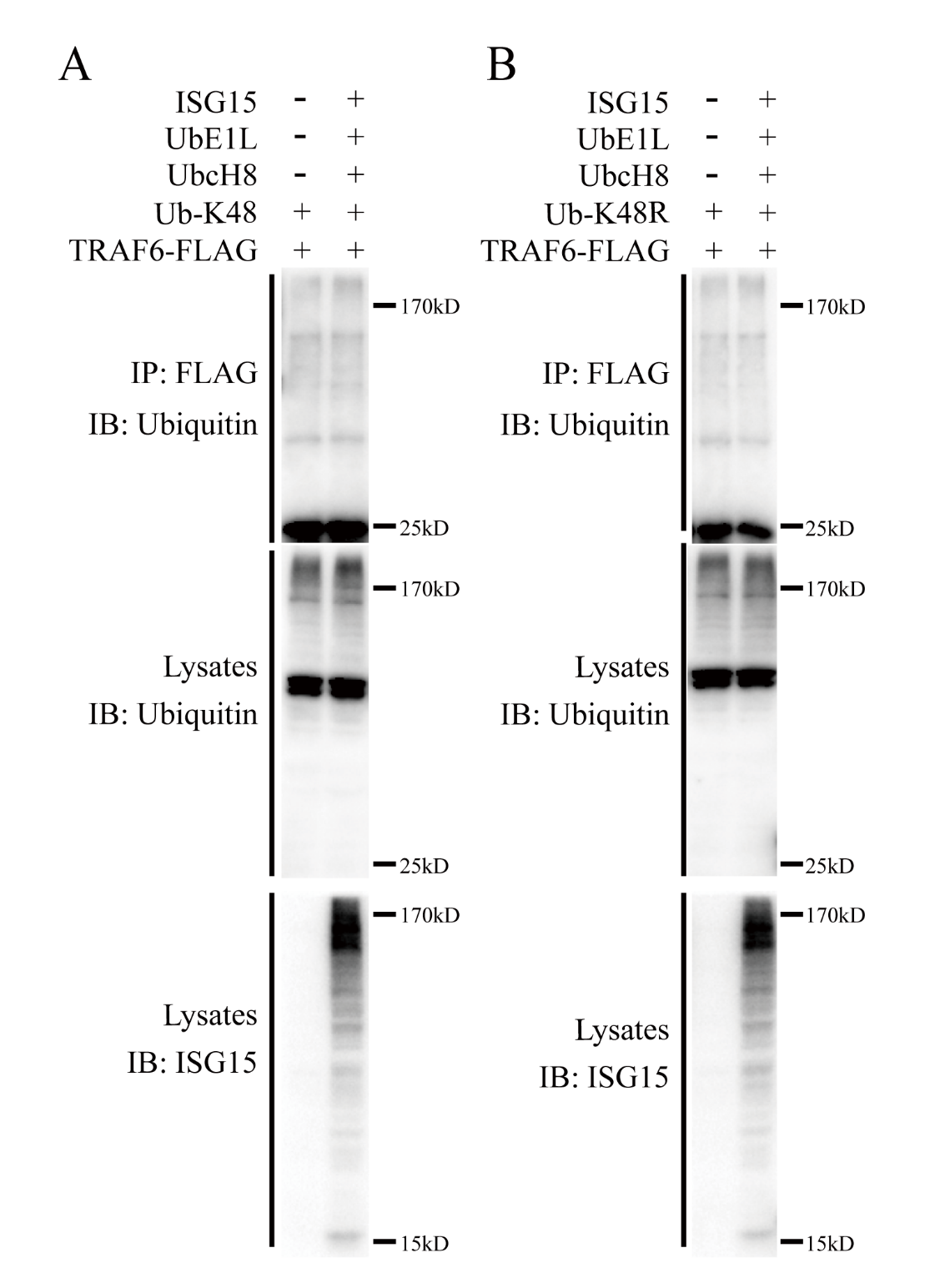


Figure S9. ISGylation did not affect K48 polyubiquitination of TRAF6. (A, B) Western blot analysis of HEK293T cells lysates co-transfected with His-tagged K48 ubiquitin/K48R ubiquitin, Flag-TRAF6 and ISGylation system (UBE1L, UbCH8 and ISG15) after immunoprecipitation with anti-Flag antibody.


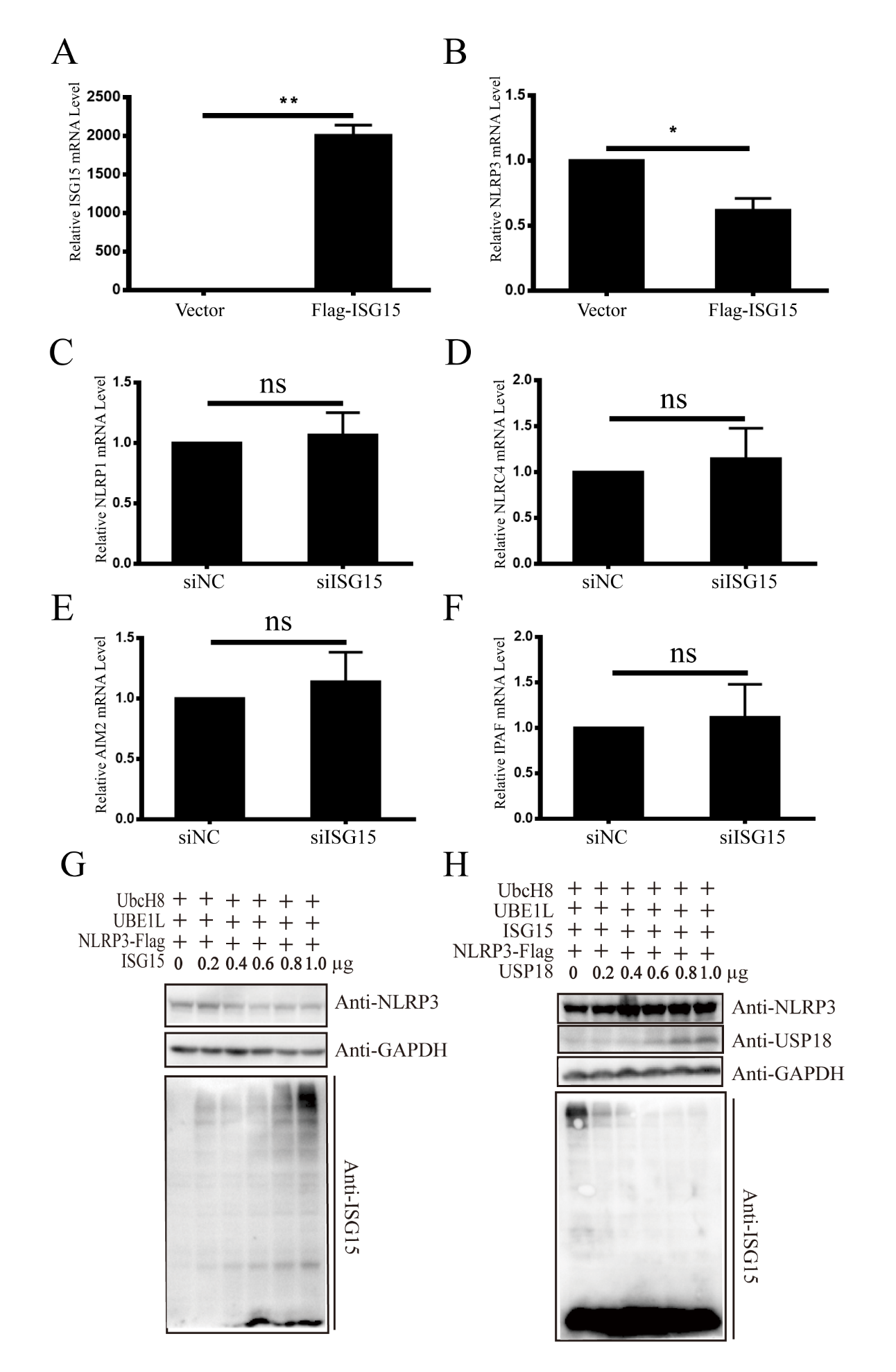


Figure S10. ISG15 negatively regulates the mRNA and protein levels of NLRP3. (A, B) Quantitative RT-PCR analysis of *ISG15*, *NLRP3* genes in U87-MG cells transfected with negative control (Vector) or Flag-ISG15. (C-F) Quantitative RT-PCR analysis of *NLRP1* (C), *NLRC4* (D), *AIM2* (E) and *IPA*F (F)gene. (G) The protein level of NLRP3 is decreased in an ISG15 concentration-dependent manner. Western blot analysis of HEK293T cells lysates co-transfected with NLRP3-Flag and ISGylation system. (H) The protein level of NLRP3 is increased in a USP18 concentration-dependent manner. Western blot analysis of HEK293T cells lysates co-transfected with NLRP3-Flag, ISGylation system and USP18. All quantitative RT-PCR experiments were repeated independently five times and all western blot analysis are repeated independently three times. Levels of significance: **P < 0.01, *P < 0.05, "ns" indicates no significant difference.

Table S1. Primer sequences used in this study

| **Gene Name** | **Sequence (5′-3′)** | **Product size (bp)** |
| --- | --- | --- |
| *ISG15*-F | CTCTGAGCATCCTGGTGAGGAA | 136 |
| *ISG15*-R | AAGGTCAGCCAGAACAGGTCGT |  |
| *IL1B*-F | CTCGCCAGTGAAATGATGGCT | 144 |
| *IL1B*-R | GTCGGAGATTCGTAGCTGGAT |  |
| *IL1R1*-F | ATGAAATTGATGTTCGTCCCTGT | 184 |
| *IL1R1*-R | ACCACGCAATAGTAATGTCCTG |  |
| *NLRP3*-F | GATTGTGGTTGGGGCGCTGTG | 120 |
| *NLRP3*-R | CGAAGTCACCGAGGGCGTTGTC |  |
| *NLRP1*-F | GCCTTCTGTGAGAGAGAGCCT | 231 |
| *NLRP1*-R | TGCAGTATGACTATGCGAGGTT |  |
| *NLRC4*-F | TGAACTGATCGACAGGATGAAC | 149 |
| *NLRC4*-R | GTCTCCAGTTTTTCAACCCAAG |  |
| *AIM2*-F | GCCTCACGTGTGTTAGATGC | 86 |
| *AIM2*-R | ATCTTCGGGGTTTCACCAGC |  |
| *IPAF*-F | AGGTCCCACAACTCGTCAAGCT | 138 |
| *IPAF*-R | TGCTCACACGATTTCCCGCCAA |  |
| *NFKB1*-F | GCAACTATGTTGGACCTGCAAA | 86 |
| *NFKB1*-R | ACCCACCAAGCTGTGAGCAT |  |
| *ACTG1*-F | CACCATTGGCAATGAGCGGTTC | 135 |
| *ACTG1*-R | AGGTCTTTGCGGATGTCCACGT |  |
| *IL6*-F | ATGAGCTCCTTCTCCACAAGCGC | 628 |
| *IL6*-R | GAAGAGCCCTCAGGCTGGACTG |  |
| *IL8*-F | ACTGAGAGTGATTGAGAGTGGAC | 112 |
| *IL8*-R | AACCCTCTGCACCCAGTTTTC |  |
| *IL18*-F | ATCGGCCTCTATTTGAAGATATGACT | 100 |
| *IL18*-R | CCTCTAGGCTGGCTATCTTTATACATACT |  |
| *IL1A*-F | CATCCTCCACAATAGCAGACAG | 105 |
| *IL1A*-R | GAGTTTCCTGGCTATGGGATAAG |  |
| *TNFA*-F | TCTCGAACCCCGAGTGACAA | 181 |
| *TNFA*-R | TGAAGAGGACCTGGGAGTAG |  |
| *IL10*-F | TGAAGAATGCCTTTAATAAGCTCCA | 135 |
| *IL10*-R | ATAGAGTCGCCACCCTGATG |  |
| *TGFB*-F | CCCACAACGAAATCTATGACAAG | 175 |
| *TGFB*-R | TATTTCTGGTACAGCTCCACGTG |  |
| *IL35*-F | CCACGTACAGGCTCGGCATGGCT | 170 |
| *IL35*-R | AGGCACGAAGCTGCTGCTGGAGC |  |
| *IL37*-F | GGACAAAGTCATCCATCCCTTC | 110 |
| *IL37*-R | GAGCCCACCTGAGCCCTATAA |  |
| *IL38*-F | CATTGAGCCTCATGCTCTGTT | 167 |
| *IL38*-R | CGCTGTCTGAGCGGATGAA |  |

Table S2. A list of full names of abbreviations used in this study.

| **Abbreviations** | **Full name** |
| --- | --- |
| ISG15 | interferon stimulated gene 15 |
| HM | human microglia |
| TRAF6 | TNF receptor associated factor 6 |
| NF-κB | nuclear factor kappa-B |
| NLRP3 | NOD-like receptor thermal protein domain associated protein 3 |
| USP18 | ubiquitin specific peptidase 18 |
| COVID-19 | Corona Virus Disease 2019 |
| SDS-PAGE | sodium dodecyl sulfate polyacrylamide gel electrophoresis |
| ELISA | Enzyme-linked Immunosorbent Assay |
| MAPKs | mitogen-activated protein kinases |
| UBE1L | Ubiquitin Activating Enzyme E1 Like Protein |
| UBCH8 | Ubiquitin/ISG15-conjugating enzyme E2 L6 |
| ISGs | interferon-stimulated genes |
| UBL | ubiquitin-like protein |
| CNS | Central Nervous System |
